# Metabolite Profile and Metabolic Network Analysis of Walnuts (*Juglans regia* L.) in Response to Chilling Stress

**DOI:** 10.3390/metabo15060394

**Published:** 2025-06-12

**Authors:** Kai Liu, Yang Li, Yaxin Sang, Yaru Zhang, Xiuhong An, Hongxia Wang, Ruifen Zhang

**Affiliations:** 1 Scientific Research Office, BinZhou Polytechnic, Binzhou 256603, China; liukai@bzpt.edu.cn (K.L.); liyang902102@163.com (Y.L.); 15615534384@163.com (Y.Z.); 2 College of Food Science and Technology, Hebei Agricultural University, Baoding 071000, China; sangyaxin@sina.com; 3 College of Horticulture, Hebei Agricultural University, Baoding 071000, China; anxiuhong2007@126.com; 4 Qingdao Academy of Agricultural Sciences, Qingdao 266000, China

**Keywords:** *Juglans regia*, chilling stress, flavonoid, metabolic network

## Abstract

Background: Walnut (*Juglans regia* L.) is a species of considerable ecological, social, and economic importance. However, comprehensive metabolomic investigations into walnut cultivars under chilling stress remain scarce. Methods: In this study, we utilized LC-MS/MS-based non-targeted metabolomics to analyze differential metabolites in two walnut cultivars exposed to chilling stress at 0.5 °C for 0 and 48 h. Results: A total of 1504 metabolites were identified, including 871 in positive ion mode and 633 in negative ion mode. Specifically, 160 and 287 differential metabolites were detected in ‘Qingxiang’ and ‘Liaoning No.8’, respectively, under positive ion mode. In negative ion mode, 83 and 206 differential metabolites were identified in ‘Qingxiang’ and ‘Liaoning No.8’, respectively. These metabolites were primarily associated with α-linolenic acid metabolism, phenylpropanoid biosynthesis, flavonoid biosynthesis, and phenylalanine metabolism, and multiple candidate genes were obtained that exhibit significant correlations with metabolites, suggesting their critical roles in the walnut’s response to chilling stress. Conclusions: This study proposes a metabolic network for walnut leaves under chilling stress, enriching our understanding of the metabolic adaptation mechanisms of walnuts to low-temperature conditions. It lays a foundation for investigating the regulatory mechanisms of metabolite synthesis under cold stress and provides important theoretical insights for breeding cold-resistant walnut cultivars.

## 1. Introduction

Temperature stress is one of the important factors affecting plant growth and development. In particular, low-temperature stress affects plant growth and development, production, and geographical distribution, resulting in significant changes in plants at the molecular, cellular, physiological, and biochemical levels [1,2,3,4] to respond to the temperature-stress environment. Plants, especially crops, are an important source of bioenergy for human survival. In total, 50% of major crops’ annual yield loss is related to abiotic stress globally, while the crops’ yield loss related to biological stresses such as diseases and insect pests is less than 20% [5]. Abiotic stresses are an important factor in agricultural production reduction [6]. Natural disasters such as cold damage, hail, drought, and waterlogging occur frequently in fruit areas in many countries [7]. Enhancing the chilling tolerance of walnuts represents a critical objective in modern breeding programs.

Walnut (*Juglans regia* L.) is a perennial tree, a nut and oil tree plant of the *Juglans* family, and has a high nutritional value, medical and health care functions, which are popular, and a very important economic value [8,9]. Walnuts are a demanding species that are not tolerant of low temperatures. They are perfectly adapted to deep, fertile, slightly acidic to slightly alkaline soil with a pH of 6.2~8.2, and the most suitable annual precipitation for walnuts is 400~1200 mm [10]. However, an unfavorable environment seriously affects the quality of walnuts. Low temperature can cause physiological changes, cell membrane damage, water metabolism disorder, and the degradation of proteins and other macromolecular substances, and it can damage the balance ability of reactive oxygen generation and removal [11]. Mild low-temperature stress can cause changes in plant leaves, stems, other morphological indicators, and biomass. This change is manifested in external morphology and internal physiological and metabolic aspects and is externally manifested as leaf wilting, yellowing, and withering, as well as a low seed setting rate [12]. In the interior, the cell membrane is destroyed, the chlorophyll content and structure are changed, etc., and severe low temperatures can cause plant metabolism disorders and even plant death [13]. Walnuts are widely distributed and planted throughout the world, covering more than forty countries and regions on six continents [14]. China’s output occupies an important position [15]. Because walnuts are a thermophilic species, their branches have a large pith, relatively high water content, and poor cold resistance. Therefore, low temperatures will affect their growth and cause economic losses in walnut cultivation [16].

Relying on the characteristics of high throughput, high sensitivity, and the wide detection range of metabolome sequencing, one can analyze plant phenotypic and physiological activity and metabolic changes caused by various external factors, which can reflect the adaptation of plants to the environment [17,18,19].

Studies have found that when the plant mesophyll tissue is developed, the thickness of the palisade tissue is large, and the arrangement is tight, which will make the leaves firmer and more resistant to harsher chilling environments [20]. Differential metabolites are mainly enriched in sugar and alcohol metabolism, amino acid metabolism, and tricarboxylic acid cycle metabolism pathways [21,22,23,24,25,26], among which proline, leucine, ornithine, and threonine can serve as the main biomarkers for the response to low-temperature stress in plants [27,28]. In *Gastrodia elata*, Zhou et al. [29] studied the effect of different temperatures on *Gastrodia elata* gene expression and metabolite content through the joint analysis of the transcriptome and metabolome; the differences were mainly distributed in seven substances, including sugar alcohols, amino acids, organic acids, lipids, nucleotide, phenolic acid, and vitamins. Zhou et al. [30] pointed out that low-temperature storage inhibited the content of abscisic acid in cantaloupe fruits but promoted the accumulation of indole acetic acid and salicylic acid. The cold resistance of ‘10–122’ was higher than that of ‘09–126’ because of changes in the content of alanine, aspartic acid, and serine. There are 323 sugars that significantly increased levels. Metabolomics is an effective method to study the response of plants to the environment [31,32,33,34] and has been widely used to study the response of plants to chilling [35,36].

Up to now, no studies have reported changes in the response of metabolites of walnuts to chilling stress. Here, an artificial incubator was used to simulate low-temperature stress in walnuts, and the metabolite changes were comprehensively analyzed using UPLC-MS/MS non-targeting technology. This study will help to better understand the metabolite changes in walnuts under low-temperature stress.

## 2. Materials and Methods

### 2.1. Plant Materials

In this investigation, annual dormant branches with uniform position, growth vigor, and diameter were collected during winter dormancy (when cold hardiness peaks in walnut plants) from four cultivars: ‘Qingxiang’, ‘Liaoning No.1’, ‘Liaoning No.7’, and ‘Liaoning No.8’. Following thorough surface cleaning, the branches were processed into 15 cm length segments. These sections underwent sequential treatment with deionized water rinses followed by moisture removal through filter paper blotting. Both cut ends were sealed with paraffin wax to prevent desiccation before subjecting the samples to controlled low-temperature treatments in programmable freezers. The experimental design incorporated differential temperature regimes: ‘Qingxiang’ and ‘Liaoning No.8’ were exposed to −20 °C and −40 °C treatments, while ‘Liaoning No.1’ and ‘Liaoning No.7’ received 0.5 °C exposure as experimental controls. All treatments were conducted for five duration intervals (12, 24, 36, 48, and 72 h) with parallel maintenance of untreated control groups at constant 0.5 °C [37].

The experimental plant materials were cultivated and maintained at Hejiang Agricultural University’s research facilities. During the 2019 growing season, uniform rootstocks established in 2018 through pot sowing of ‘zanmei’ walnut seeds were grafted with two cultivars: ‘Liaoning No.8’ and ‘Qingxiang’. For controlled low-temperature exposure, selected seedlings were transferred to a precision climate-controlled chamber where they underwent a 48-h continuous 0.5 °C cold stress treatment, with parallel control groups maintained under ambient conditions. Following the treatment protocol, mature leaves from both cultivars were systematically harvested, flash-frozen in liquid nitrogen within 30 s of collection, and subsequently stored in an ultra-low temperature freezer (−80 °C) to preserve metabolic integrity prior to phytochemical analysis.

### 2.2. REC Determination

Three branches were randomly sampled from each of the four walnut cultivars (‘Qingxiang’, ‘Liaoning No.8’, ‘Liaoning No.7’, and ‘Liaoning No.1’). Following triple rinsing with deionized water, the samples were surface-dried using sterile gauze and filter paper. Branches were subsequently sectioned into 0.5-cm segments, with approximately 0.5 g of tissue precisely weighed and transferred to Erlenmeyer flasks containing 40 mL deionized water. All treatments were performed in triplicate.

The samples underwent sequential processing as follows:

(1) 15-min boiling with subsequent sealing using parafilm
(2) Continuous extraction in a temperature-controlled shaker (25 °C, 15 h)
(3) Initial conductivity measurement (A1) using a DDSJ-307F conductivity meter (Shanghai Yidian Science Instrument Co., Ltd., Shanghai, China)
(4) Thermal treatment in an 85 °C water bath for 20 min to achieve complete cellular disruption
(5) Post-extraction conductivity measurement at 25 °C (A2)

Throughout the experimental process, parafilm sealing was maintained to prevent solvent evaporation and ensure constant solution volume. Relative electrolyte leakage was calculated using the formula: Relative conductivity = A1/A2 × 100%.

### 2.3. Metabolites’ Extraction

#### 2.3.1. Tissue Sample Processing

(1) Homogenization: Approximately 100 mg of tissue specimen was cryogenically pulverized in liquid nitrogen using a mortar and pestle.
(2) Extraction: The resulting homogenate was vortex-resuspended in 1 mL of pre-chilled 80% (*v*/*v*) methanol aqueous solution and maintained at 4 °C for 5 min with ice-bath incubation.
(3) Primary Centrifugation: Cellular debris was removed by centrifugation at 15,000× *g* (4 °C, 20 min) using a refrigerated centrifuge.
(4) Dilution: The methanolic supernatant was diluted with LC-MS grade water to achieve a final methanol concentration of 53% (*v*/*v*).
(5) Secondary Clarification: The diluted solution underwent additional centrifugation under identical parameters (15,000× *g*, 4 °C, 20 min) in sterile microcentrifuge tubes.
(6) LC-MS/MS Analysis: The clarified supernatant was directly injected into the LC-MS/MS system for metabolite profiling [38].

#### 2.3.2. Quality Control (QC) Samples

Pooled QC samples were generated by combining equal-volume aliquots from individual experimental samples prior to instrumental analysis.

#### 2.3.3. Method Blank Preparation

Blank controls were processed in parallel using 53% (*v*/*v*) methanol aqueous solution instead of biological material, following identical processing conditions to monitor potential background interference.

### 2.4. Instrument Parameters

#### 2.4.1. Chromatographic Conditions (Vanquish UHPLC, Thermo Fisher, Dreieich, Germany)

Column: HypesilGoldcolumn (C18);

Column temperature: 40 °C;

Flow rate: 0.2 mL/min;

Positive mode—mobile phase A: 0.1% formic acid;

Mobile phase B: methanol;

Negative mode—mobile phase A: 5 mM ammonium acetate, pH 9.0;

Mobile phase B: methanol;

Chromatographic gradient elution program:
**Time****A%****B%**09821.598212010014010014.198217982


#### 2.4.2. Mass Spectrometry Conditions (Q Exactive™ HF, Thermo Fisher, Dreieich, Germany)

The mass spectrometry analysis was performed with a full scan range of *m*/*z* 100–1500. Electrospray ionization (ESI) parameters were optimized as follows: spray voltage maintained at 3.2 kV, sheath gas flow rate set to 40 arb., auxiliary gas flow rate at 10 arb., and capillary temperature stabilized at 320 °C. Both positive and negative ionization modes were alternately applied during acquisition. Data-dependent acquisition (DDA) mode was employed for MS/MS analysis, automatically triggering secondary fragmentation scans based on precursor ion intensity thresholds.

### 2.5. Methods of Qualitative and Quantitative Analysis of Metabolites

Metabolite identification was performed through a two-tiered analytical approach. Initially, molecular weights were calculated based on the mass-to-charge ratio (*m*/*z*) of precursor ions detected in primary MS spectra. Putative molecular formulas were generated through computational analysis incorporating mass accuracy (≤5 ppm deviation) and adduct ion patterns. These preliminary identifications were then cross-referenced with established spectral libraries containing experimental MS/MS data.

For structural verification, experimental MS/MS spectra were matched against reference databases using dual validation criteria: (1) fragment ion congruence (*m*/*z* matching within 5 ppm tolerance) and (2) collision energy-dependent fragmentation patterns. To ensure analytical consistency, metabolites demonstrating intra-batch reproducibility with a coefficient of variation (CV) <30% in quality control samples (based on established criteria [39]) were retained for downstream analyses. Chromatographic data processing was conducted using Compound Discoverer 3.1 software. Peak integration parameters were optimized to achieve consistent quantification through total ion current normalization. Relative metabolite abundances were calculated from normalized peak areas of characteristic ions, with these standardized quantitative values forming the basis for subsequent multivariate analyses.

### 2.6. Data Quality Assessment

Quality control (QC) samples were implemented throughout the analytical workflow to ensure system reliability and data reproducibility. Prior to sample analysis, QC preparations were utilized to verify instrument performance status and equilibrate the LC-MS system. During the experimental sequence, ten QC aliquots were intermittently inserted at equidistant intervals to monitor longitudinal system stability and facilitate batch-to-batch normalization. Following the completion of sample analyses, three additional QC sets were analyzed in segmented batches to establish analytical variation thresholds. The acquired MS/MS spectra from experimental samples were subsequently subjected to metabolite identification through database matching against standardized spectral libraries.

### 2.7. OPLS-DA Analysis

For multivariate analysis, we processed the raw metabolomics data using the metaX platform [40], which enabled preprocessing and subsequent partial least squares-discriminant analysis (PLS-DDA) to determine variable importance in projection (VIP) scores for each metabolite. In parallel, univariate statistical analysis was performed through two complementary approaches: (1) Student’s *t*-test was applied to assess intergroup differential significance (*p*-values), and (2) fold change (FC) values were calculated to quantify relative concentration differences between experimental groups.

### 2.8. Differential Metabolite Analysis

The identification of differential metabolites was conducted through a comprehensive evaluation of three key statistical parameters: variable importance in projection (VIP), fold change (FC), and statistical significance (*p*-value). Specifically, VIP values were derived from the partial least squares-discriminant analysis (PLS-DA) model, representing the contribution weight of each metabolite in the first principal component [41]. FC was calculated as the ratio of mean metabolite concentrations between comparative groups across all biological replicates. Statistical significance was determined using Student’s *t*-test [4], with *p*-values indicating the probability of observed differences occurring by chance.

To ensure rigorous selection, we established the following thresholds: VIP > 1.0 (indicating substantial contribution to group separation), absolute log_2_FC > 0.585 (equivalent to FC > 1.5 or <0.667), and *p*-value < 0.05 [42]. Metabolites satisfying all three criteria were considered statistically significant differential metabolites.

### 2.9. Statistical Analysis of Data

Visualization and statistical analyses were performed using R programming language (v4.2.2). The volcano plot was generated using the ggplot2 package (v3.4.2) to visualize metabolite significance by integrating three key parameters: variable importance in projection (VIP) scores, log2-transformed fold change values, and −log10-transformed *p*-values. Differential metabolites were identified using threshold criteria of VIP > 1.0, absolute log2FC ≥ 1.0, and *p* < 0.05.

Pearson correlation analysis between metabolites was conducted using R’s built-in cor() function, with statistical significance determined through permutation testing (cor.mtest() function, 1000 iterations). Significant correlations (*p* < 0.05) were visualized using the corrplot package (v0.92), with hierarchical clustering applied to reveal association patterns.

Functional annotation of metabolites was performed through KEGG pathway analysis (KEGG REST API, October 2022 release). Pathway enrichment was assessed using ggplot2-generated bubble charts, with enrichment significance determined by two criteria: (1) pathway component ratio (x/n) exceeding background ratio (y/n), and (2) Benjamini–Hochberg adjusted *p*-value < 0.05.

Raw sequencing data have been deposited in the National Genomics Data Center (NGDC; https://ngdc.cncb.ac.cn/) under BioProject accession PRJCA006073. Processed data and analysis scripts are available through the corresponding author upon reasonable request.

## 3. Results

### 3.1. Relative Electrical Conductivity Changes in Walnut in Response to Chilling Stress

**Figure 1:**
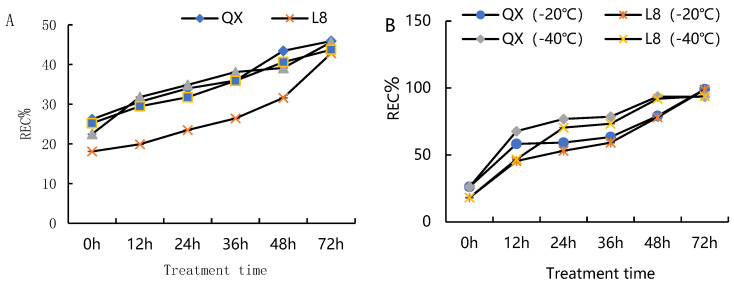
The effect of cold stress on the electrical conductivity of walnut branches. (**A**) The effect of chilling stress on four different walnut branches at different times; (**B**) the effect of different cold stress temperatures on two different walnut branches.

The REC (relative electrical conductivity) of the branches of the four cultivars increased significantly under low-temperature stress (Figure 1A). At 0.5 °C, the REC of the two cultivars ‘Liaoning NO.8’ and ‘Qingxiang’ was significantly different before 48 h of chilling stress; the REC after 72 h in the four varieties showed no significant difference. The difference between ‘Qingxiang’, ‘Liaoning NO.1’, and ‘Liao NO.7’ was only significant at 48 h but not significant at other times. This indicated that chilling stress caused damage to the cell membranes of the four walnut varieties; the electrical conductivity showed that ‘Liaoning NO.8’ was tolerant to cold stress and had lower ion permeability than the other three varieties. Then, we selected the different levels of change in the REC of ‘Qingxiang’ and ‘Liaoning NO.8’ after stress at −20 °C and −40 °C for times of 0, 12, 24, 36, 48, and 72 h (Figure 1B). We found that the change trends of the two varieties at −20 °C and −40 °C were consistent. After 36 h, the changes in the REC were not significant. At −20 °C, the difference between the two cultivars under chilling stress for 12 h was the most significant. Over time, the relative conductivity gap of the two cultivars gradually narrowed until it was insignificant at −40 °C; the REC at −40 °C was higher than the treatment at −20 °C for the same time. Therefore, we selected the leaf materials of ‘Qingxiang’ and ‘Liaoning NO.8’ at 0.5 °C for 48 h for the identification of non-target metabolites.

### 3.2. Qualitative and Quantitative Analysis of Metabolites

**Figure 2:**
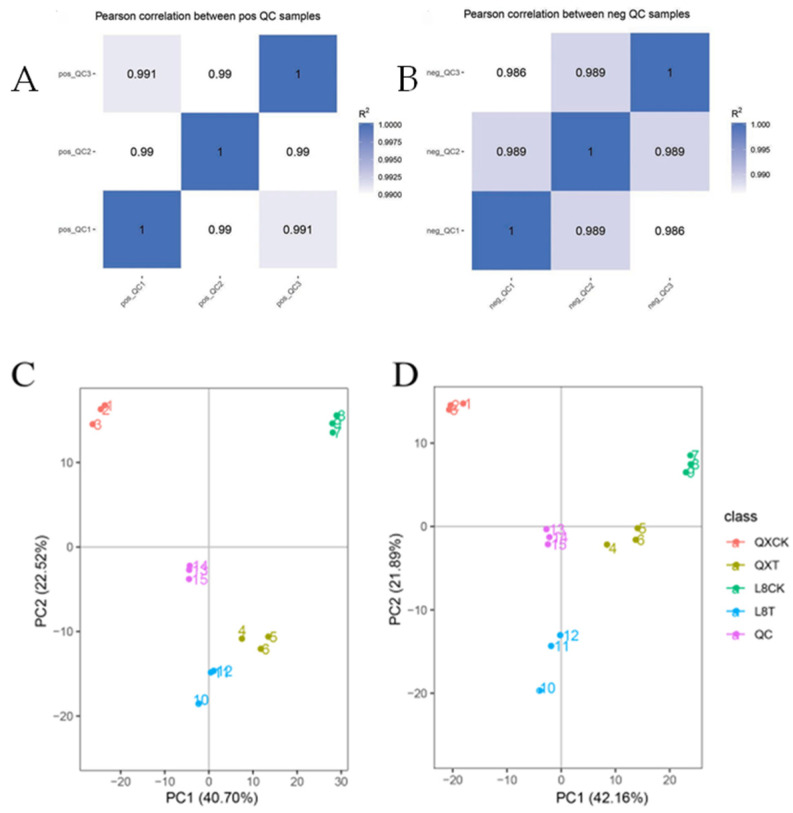
QC sample correlation analysis and PCA analysis of the total sample. (**A**) Correlation analysis in positive ion mode; (**B**) correlation analysis in negative ion mode; (**C**) PCA analysis in positive ion mode; (**D**) PCA analysis in negative ion mode.

The higher the correlation (Figure 2) of the QC samples (the closer R^2^ is to 1), the better the stability of the whole detection process and the higher the data quality.

The peaks extracted from all the experimental samples and the QC samples were subjected to UV calibration (univariate calibration, univariate standardization); then, PCA analysis was performed (Figure 2). As shown in Figure 2, the distribution of the QC samples was close, and the difference between the QC samples was small. The stability of the whole method was good, and the data quality was high. Hence, the test material data were credible.

**Figure 3:**
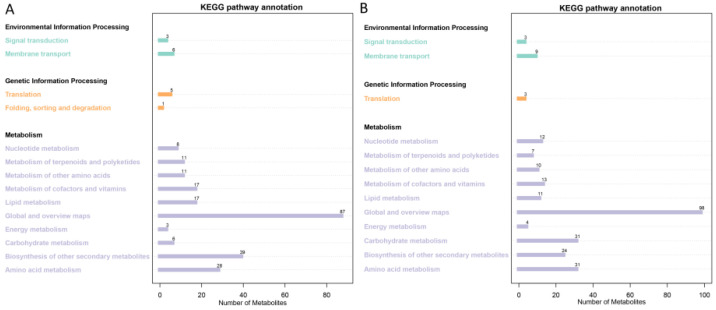
KEGG pathway annotation. (**A**) Positive ion mode; (**B**) negative ion mode.

**Figure 4:**
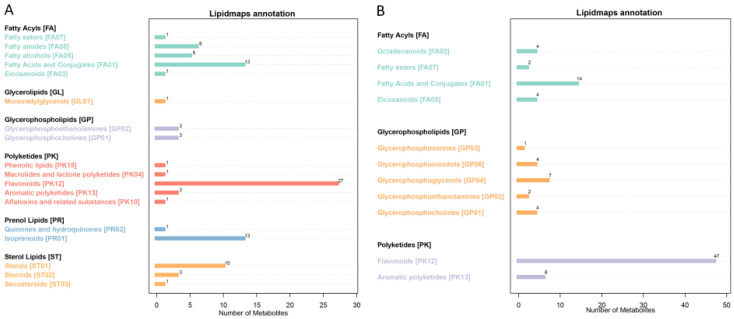
LIPID MAPS classification annotation. (**A**) Positive ion mode; (**B**) negative ion mode. Note: the abscissa represents the number of metabolites, and the ordinate represents the annotated LIPID MAPS lipid classification.

The main databases include KEGG and LIPID MAPS. We annotated the identified metabolites using these databases to understand the functional characteristics and classification of different metabolites. The KEGG PATHWAY database is a collection of metabolic pathways, which mainly divides the biological metabolic pathways into seven categories: metabolism, genetic information processing, environmental information processing, cellular processes, organic systems, and drug development. Pathway analysis can determine the most important biochemical metabolic pathways and signal transduction pathways involved in metabolite changes. Using non-targeted metabolomics, 1504 metabolites were identified, including 871 positive ion metabolites and 633 negative ion metabolites. KEGG annotated 284 positive ion metabolites and 242 negative ion metabolites (Figure 3), including signal transduction (3), membrane transport (9), translation (3), and nucleotide metabolism (12) in negative ion mode, as well as signal transduction (3), membrane transport (6), lipid metabolism (17), biosynthesis of other secondary metabolites (39), and amino acid metabolism (28) in positive ion mode. We annotated 94 positive ion metabolites and 115 negative ion metabolites (Figure 4) using LIPID MAPS. Positive ion metabolites included aflatoxins and related substances (1), aromatic polyketides (3), eicosanoids (1), and monoradylglycerols (1); in negative ion mode, there were aromatic polyketides (6), bile acids and derivatives (1), eicosanoids (4), fatty acids and conjugates (14), fatty esters (2), and flavonoids (47), among others.

### 3.3. PLS-DA Analysis

**Figure 5:**
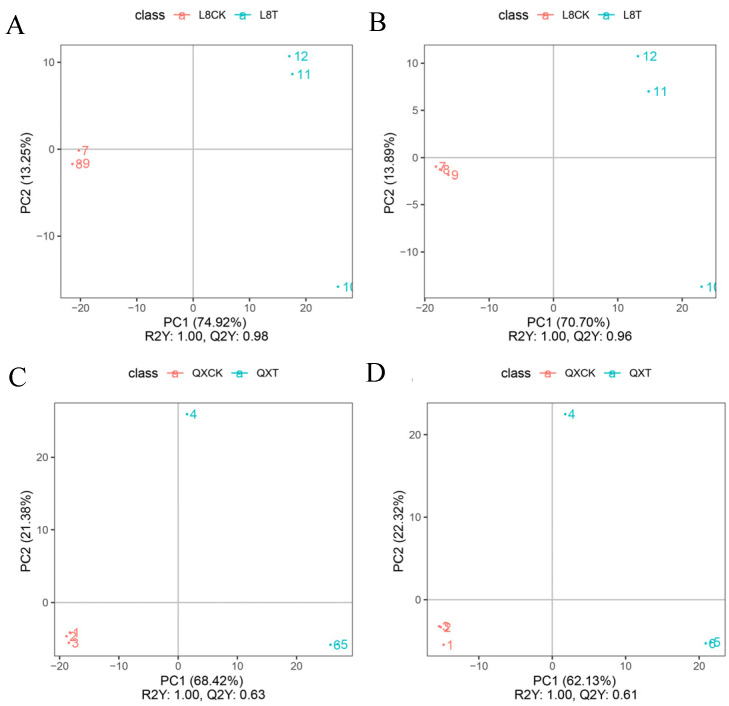
Orthogonal partial least squares discriminant analysis (OPLS-DA) scores. (**A**) Positive ion mode in ‘Liao NO.8’; (**B**) negative ion mode in ‘Liao NO.8’; (**C**) positive ion mode in ‘Qingxiang’; (**D**) negative ion mode in ‘Qingxiang’. R^2^Y scores and Q^2^ values represent the interpretation rate of the model to the Y matrix and the prediction ability of the model, respectively. When Q^2^ > 0.5, the model can be considered an effective model, and Q^2^ > 0.9.

PLS-DA (partial least squares discriminant analysis) is a supervised statistical method of discriminant analysis. The results showed that the R2Y scores were all higher than 0.99, the Q^2^ scores were all higher than 0.6 (Figure 5), and the Q^2^ scores were all higher than 0.9 in ‘Liaoning NO.8’. It was an excellent model, which confirmed that the differential metabolites were responsive to cold stress.

### 3.4. Differential Metabolites’ Screening

**Figure 6:**
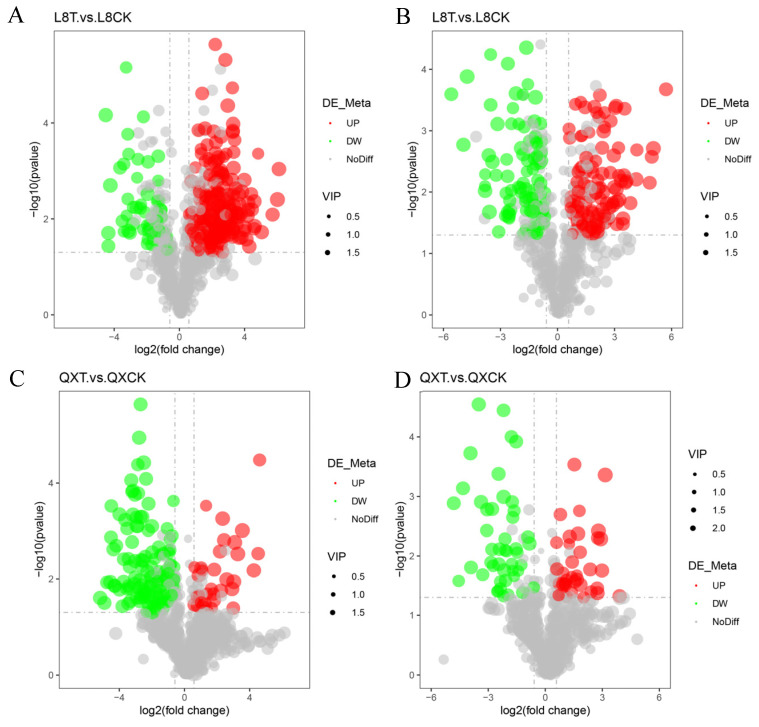
Volcano map of differential metabolites. Note: each dot represents a metabolite: the significantly upregulated metabolites are represented by red dots, the significantly downregulated metabolites are represented by green dots, and the size of the dot represents the VIP value. (**A**) Positive ion mode in ‘Liaoning NO.8’; (**B**) negative ion mode ‘in Liaoning NO.8’; (**C**) positive ion mode in ‘Qingxiang’; (**D**) negative ion mode in ‘Qingxiang’.

The screening of differential metabolites mainly involves three parameters: VIP, FC, and *p*-value. The threshold was set to VIP > 1.0, FC > 1.5, or FC < 0.667, and *p*-value < 0.05, and the selected differential metabolites are shown in Table 1 and Figure 6. Using non-targeted metabolomics, 1504 metabolites were identified, including 871 positive ion metabolites and 633 negative ion metabolites. After 48 h of chilling stress for ‘Qingxiang’, 160 positive ion mode metabolites changed significantly; of these, 32 significantly increased, and 127 significantly decreased. Further, 83 metabolites in negative ion mode changed significantly; of these, 37 were significantly increased, and 46 decreased significantly. In ‘Liao NO.8’, after cold stress for 48 h, 287 positive ions changed significantly; of these, 233 significantly increased, and 54 significantly decreased. Further, 206 negative ion mode metabolites changed slightly; of these, 114 metabolites significantly increased, and 92 metabolites significantly decreased.

**Figure 7:**
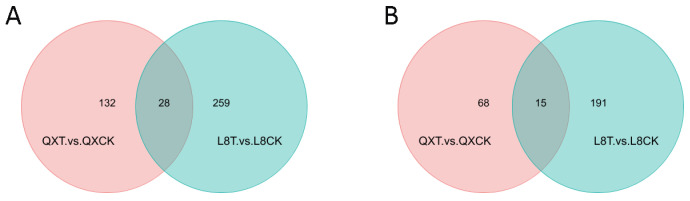
Venn map of differential metabolites. (**A**) Positive ion mode; (**B**) negative ion mode.

From the Venn diagram of differential metabolites (Figure 7), for the differential metabolites of the two varieties of ‘Qingxiang’ and ‘Liaoning No.8’ after 48 h of cold stress in positive ion mode, 28 metabolites co-existed, with 132 significant differences in ‘Qingxiang’ and 259 significantly different in ‘Liaoning No.8’; in negative ion mode, 15 metabolites co-existed, with 68 species significantly different in ‘Qingxiang’ and 191 species significantly different in ‘Liaoning No.8’.

**Figure 8:**
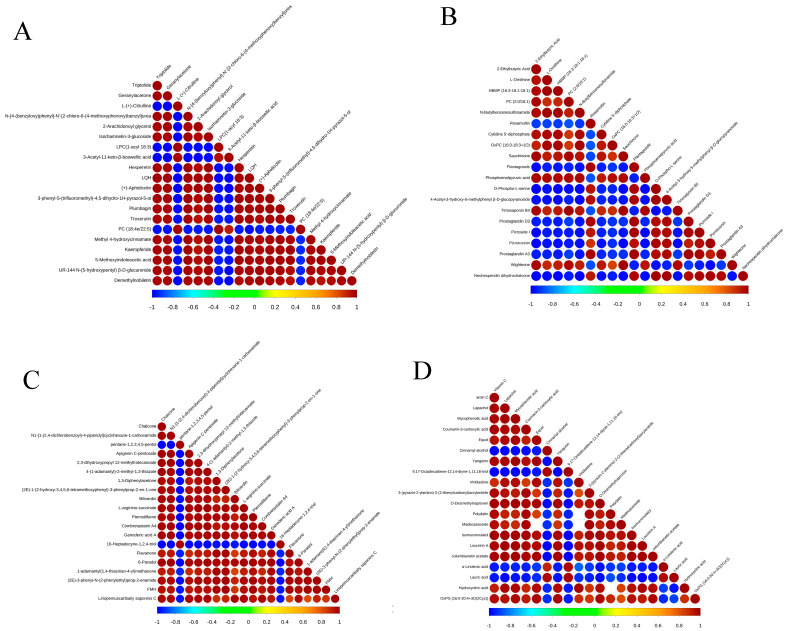
Correlation diagram of different metabolites. (**A**) Positive ion mode in ‘Liao NO.8’; (**B**) negative ion mode in ‘Liao NO.8’; (**C**) positive ion mode in ‘Qingxiang’; (**D**) negative ion mode in ‘Qingxiang’. Note: The highest correlation is 1, which is a complete positive correlation (red), and the lowest correlation is −1, which is a complete negative correlation (blue). The part with no color indicates a *p*-value > 0.05. The figure shows the correlation of the Top20 differential metabolites sorted by *p*-value from small to large.

Different metabolites have a synergistic or mutually exclusive relationship. The purpose of the correlation analysis of differential metabolites is to check the consistency of the metabolites and the metabolite change trends. When the linear relationship between two metabolites increases, the positive correlation tends to 1 and the negative correlation to −1. Figure 8 shows the correlation of the Top20 differential metabolites sorted by *p*-value from small to large. 2-ethylbutyric acid has a positive correlation with L-ornithine, HBMP, PC, N-butylbenzenesulfonamide, etc., and it has a negative correlation with rosamultin, plantagoside, O-phospho-L-serine, etc. Correlations between different substances may be due to close links between the substances.

### 3.5. KEGG Enrichment Result Analysis

**Figure 9:**
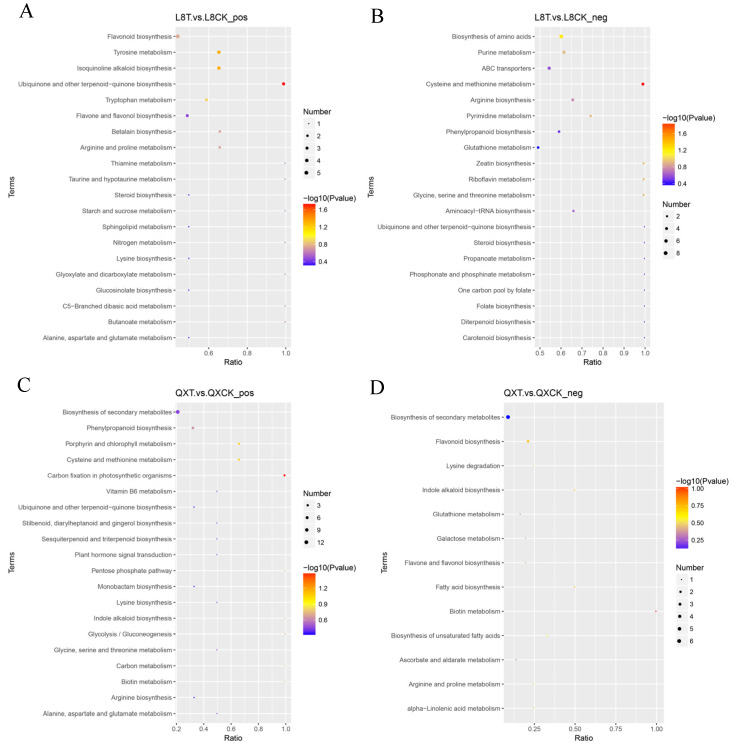
Bubble chart of KEGG enrichment. (**A**) Positive ion mode in ‘Liao NO.8’; (**B**) negative ion mode in ‘Liao NO.8’; (**C**) positive ion mode in ‘Qingxiang’; (**D**) negative ion mode in ‘Qingxiang’. Note: The abscissa in the figure is x/y (the number of differential metabolites in the corresponding metabolic pathway/the total number of metabolites identified in the pathway). The larger the value, the higher the enrichment of differential metabolites in the pathway. The color of the dot represents the *p*-value of the hypergeometric test. The smaller the value, the greater the reliability of the test and the more statistically significant it is. The size of the dot represents the number of different metabolites in the corresponding pathway. The larger the dot, the more differential metabolites in the pathway.

The enrichment result takes the KEGG pathway as the unit, and the hypergeometric test is applied, as shown in the figure below. After 48 h of cold stress in ‘Liao 8’, the pathway in positive ion mode (Figure 9A) was mainly enriched in flavonoid biosynthesis, isoquinoline alkaloid biosynthesis, tyrosine metabolism, ubiquinone and other terpenoid−quinone biosynthesis, flavonol and flavonol biosynthesis, etc. Under negative ion mode (Figure 9B), it was mainly enriched in the biosynthesis of amino acids, cysteine and methionine metabolism, phenylpropanoid biosynthesis, arginine biosynthesis, etc. Under chilling stress for 48 h in ‘Qingxiang’, in positive ion mode (Figure 9C), the pathway was mainly enriched in the biosynthesis of secondary metabolites, phenylpropanoid biosynthesis, carbon fixation in photosynthetic organisms, glycolysis/gluconeogenesis, etc. In negative ion mode (Figure 9D), it was mainly enriched in the biosynthesis of secondary metabolites, biosynthesis of unsaturated fatty acids, alpha-linolenic acid metabolism, etc.

### 3.6. Analysis of Comprehensive Metabolomics and Transcriptomics Networks Under Chilling Stress

**Figure 10:**
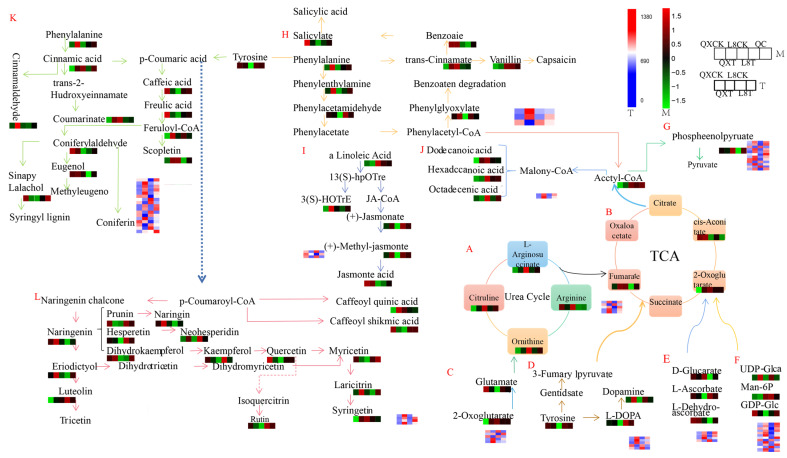
Analysis of metabolic networks in the leaves of walnuts under chilling stress. The differential metabolite (M) changes were represented by the log2 ratio. Blue represents a decrease in content, and red represents an increase in content. Transcriptional expression (T) was expressed as FPKM. The deeper the red, the higher the transcription level, and the deeper the blue, the lower the transcription level. (**A**) Urea cycle; (**B**) citrate cycle; (**C**) arginine biosynthesis; (**D**) tyrosine metabolism; (**E**) ascorbate and aldarate metabolism; (**F**) amino sugar and nucleotide sugar metabolism; (**G**) glycolysis/gluconeogenesis; (**H**) phenylalanine metabolism; (**I**) α-linolenic acid metabolism; (**J**) fatty acid biosynthesis; (**K**) phenylpropanoid biosynthesis; (**L**) flavonoid biosynthesis. M: metabonomics; T: transcriptome.

To comprehensively elucidate the metabolic alterations in walnuts under chilling stress, we constructed a metabolic pathway framework based on the existing literature and a web-based metabolic pathway database. The key pathways identified include the tricarboxylic acid (TCA) cycle, urea cycle, phenylpropanoid biosynthesis pathway, unsaturated fatty acid biosynthesis pathway, and flavonoid synthesis pathway (Figure 10).

We identified 62 metabolites across 12 metabolic pathways, with flavonoid biosynthesis being the most prominent. Following 48 h of cold stress in ‘Qingxiang’ walnut, significant increases were observed in cinnamic acid, methyl dihydrojasmonate, L-phenylalanine, fumaric acid, naringenin, cinnamaldehyde, phenethylamine, 13(S)-HOTrE, palmitic acid, dopamine, and luteolin. These metabolites were primarily concentrated in α-linolenic acid metabolism, phenylpropanoid biosynthesis, and flavonoid biosynthesis pathways. In contrast, quercetin, myricetin, kaempferol, ferulic acid, caffeic acid, and salicylic acid exhibited significant declines, which were also mainly associated with phenylalanine metabolism, phenylpropanoid biosynthesis, and flavonoid biosynthesis. Pathway analysis revealed that these changes were linked to the walnut’s response to cold stress, with the latter pathways being more active. The reduction in certain metabolites appears to enhance the content of others, thereby improving the cold tolerance of walnuts.

In ‘Liao No.8’ walnuts subjected to 48 h of cold stress, phenethylamine, luteolin, ferulic acid, caffeic acid, phenylacetaldehyde, 5-O-caffeoylshikimic acid, ascorbic acid, L-glutamic acid, methyl dihydrojasmonate, myricetin, and kaempferol showed significant increases, primarily concentrated in the phenylpropanoid biosynthesis and flavonoid biosynthesis pathways. Conversely, elaidic acid, dehydroascorbic acid, citrulline, alpha-ketoglutaric acid, palmitic acid, fumaric acid, L-ornithine, and naringenin exhibited significant decreases, also mainly concentrated in the phenylpropanoid biosynthesis and flavonoid biosynthesis pathways.

Due to differences in the low-temperature tolerance between the two walnut varieties, certain metabolites displayed contrasting trends. For example, alpha-ketoglutaric acid, palmitic acid, fumaric acid, phosphoenolpyruvic acid, and dodecanedioic acid increased in ‘Qingxiang’ but decreased in ‘Liao No.8’ under cold stress. Similarly, caffeic acid, ascorbic acid, L-glutamic acid, myricetin, and kaempferol increased in ‘Liao No.8’ but decreased in ‘Qingxiang’. However, some metabolites exhibited consistent trends, such as methyl dihydrojasmonate, luteolin, phenethylamine, and dopamine, which significantly increased in both varieties under cold stress, while elaidic acid, dehydroascorbic acid, and phosphoenolpyruvic acid significantly decreased.

These metabolite changes reflect the walnut’s adaptive response to cold stress. Integrated with transcriptome data analysis, 74 differentially expressed genes were identified across the 12 pathways, including 17 genes specifically involved in the phenylpropanoid biosynthesis pathway. This comprehensive analysis provides insights into the molecular mechanisms underlying walnut cold tolerance.

In the four highly active pathways—α-linolenic acid metabolism, phenylpropanoid biosynthesis, flavonoid biosynthesis, and phenylalanine metabolism—we conducted a correlation analysis between the differentially expressed genes (DEGs) and differential metabolites. The results revealed several significant correlations (Table 2, Table 3, Table 4 and Table 5) as follows:

- 109013393 was significantly negatively correlated with methyl dihydrojasmonate and methyl jasmonate (Table 2).
- 109018148 was significantly positively correlated with L-phenylalanine and phenethylamine (Table 3).
- 109010746 was significantly positively correlated with laricitrin (Table 4).
- 108993196 was significantly positively correlated with neohesperidin and ferulaldehyde (Table 4).
- 108989769 was positively correlated with neohesperidin, rutin, and hesperetin and significantly negatively correlated with eugenol (Table 4).
- 109020389 was significantly positively correlated with L-phenylalanine and ferulaldehyde (Table 5).
- 109020701 was highly significantly positively correlated with L-phenylalanine and significantly positively correlated with ferulaldehyde (Table 5).
- 109003193 was significantly positively correlated with coniferin (Table 5).
- 108999621 and 109004171 were significantly negatively correlated with ferulaldehyde (Table 5).
- 109009576 and 108983554 were significantly positively correlated with ferulaldehyde, with the latter also showing a significant positive correlation with L-phenylalanine (Table 5).
- 109005199 was significantly positively correlated with coniferin (Table 5).
- 108996382 was significantly negatively correlated with L-phenylalanine and ferulaldehyde (Table 5).

These findings provide valuable insights into the regulatory relationships between genes and metabolites, offering a theoretical foundation for further research on gene-mediated metabolic regulation in response to cold stress.

**Table 2:** Correlation analysis between DEGs and metabolism in α-linolenic acid metabolism.

	id	109013393	109002743	108997763
	
Linoleic acid		0.483	−0.341	0.264
13(S)-HOTrE		−0.236	−0.035	−0.273
Jasmonic acid		−0.15	0.251	0.069
Methyl dihydrojasmonate		−0.683 *	0.13	−0.568
Methyl jasmonate		−0.582 *	0.175	−0.545

Note: * *p* < 0.05.

**Table 3:** Correlation analysis between DEGs and metabolism in phenylalanine metabolism.

	id	109018148	108987596	109009407
	
Salicylic acid		−0.268	−0.227	0.042
L-phenylalanine		0.690 *	0.544	−0.356
Phenethylamine		0.756 **	0.52	−0.347
Phenylacetaldehyde		0.059	0.559	−0.225
Phenylglyoxylic acid		0.307	0.24	−0.026
Trans-cinnamic acid		0.357	0.209	−0.131
Vanillin		−0.243	0.036	0.182
Benzoic acid		0.35	0.078	−0.17

Note: * *p* < 0.05, ** *p* < 0.01.

**Table 4:** Correlation analysis between DEGs and metabolism in flavonoid biosynthesis.

	id	109010746	108993196	108989769	108997708
	
1,3-Dicaffeoylquinic acid		0.529	0.202	−0.032	0.432
Myricetin		−0.125	0.198	0.446	−0.105
Laricitrin		0.619 *	0.498	−0.061	0.377
Syringetin		0.138	−0.018	0.07	−0.002
Naringin		0.463	0.026	−0.163	0.29
Naringenin		0.492	−0.013	−0.264	0.283
Luteolin		0.079	0.332	0.559	0.229
5-O-Caffeoylshikimic acid		−0.213	−0.047	−0.012	−0.164
Neohesperidin		0.381	0.806 **	0.695 *	0.512
Kaempferol		0.133	0.437	0.43	−0.058
Quercetin		−0.124	−0.106	−0.002	−0.182
Rutin		−0.103	0.511	0.616 *	−0.054
Prunin		−0.349	0.014	0.423	−0.301
Hesperetin		0.055	0.535	0.629 *	0.179
Dihydrokaempferol		0.412	−0.132	0.195	0.19
Eriodictyol		0.432	−0.028	−0.174	0.306

Note: * *p* < 0.05, ** *p* < 0.01.

**Table 5:** Correlation analysis between DEGs and metabolism in phenylpropanoid biosynthesis.

	id	109020389	108993196	109020701	109003193	108999621	109009576	109004171	108983554	109005199	108996382	108989769
	
L-phenylalanine		0.890 **	0.466	0.712 **	0.155	−0.414	0.37	−0.487	0.802 **	0.124	−0.696 *	0.282
Trans-cinnamic acid		0.36	−0.137	0.231	−0.432	0.15	−0.164	0.215	0.24	−0.417	−0.266	−0.231
Cinnamaldehyde		0.566	0.434	0.536	0.18	−0.357	0.483	−0.174	0.366	0.177	−0.255	0.068
Ferulaldehyde		0.811 **	0.638 *	0.639 *	0.139	−0.599 *	0.615 *	−0.721 **	0.796 **	0.036	−0.631 *	0.549
Eugenol		0.164	−0.428	0.038	0.158	−0.095	−0.433	0.305	0.209	0.224	−0.102	−0.607 *
Coniferin		0.158	−0.046	0.166	0.614 *	−0.243	−0.003	−0.165	0.223	0.659 *	−0.18	−0.173
Caffeic acid		−0.331	−0.183	−0.233	0.499	−0.058	−0.12	−0.057	−0.157	0.477	0.16	−0.074
Ferulic acid		−0.42	−0.308	−0.318	0.507	−0.063	−0.248	0.076	−0.236	0.557	0.244	−0.246
Sinapyl alcohol		−0.094	−0.236	0.155	0.16	−0.176	−0.098	0.087	−0.222	0.197	0.256	0.131
Scopoletin		0.469	−0.162	0.226	−0.258	0.031	−0.274	0.102	0.406	−0.196	−0.37	−0.277
Scopolin		−0.112	−0.244	−0.216	0.457	−0.149	−0.352	0.033	0.047	0.548	0.058	−0.283
Cinnamic acid		0.428	−0.014	0.233	−0.372	−0.006	−0.165	0.067	0.332	−0.305	−0.295	−0.023

Note: * *p* < 0.05, ** *p* < 0.01.

## 4. Discussion

Chilling stress, particularly late spring cold and late frost, represents one of the primary environmental factors that disrupt the normal growth and development of plants. Exposure to temperature stress can impair enzyme activity, leading to alterations in metabolic rates and networks associated with critical processes such as photosynthesis, respiration, and protein synthesis. Metabolomics offers a comprehensive approach to analyzing the dynamic changes in metabolites, thereby elucidating the mechanisms underlying temperature stress responses [36]. The structure and properties of biofilms are closely linked to plant cold resistance, with the plasma membrane being the primary site of chilling injury [43]. In this study, the ion permeability or relative electrical conductivity (REC) of walnut leaves gradually increased following chilling stress, indicating varying degrees of damage to the leaf biofilm. The content of unsaturated fatty acids in membrane lipids is strongly correlated with a plant’s ability to tolerate chilling stress. An increase in unsaturated fatty acid content can reduce membrane rigidity and enhance fluidity, thereby promoting stability at low temperatures and improving cold resistance [44,45,46]. Sun et al. [47] demonstrated that under low-temperature stress, the contents of linoleic acid and linolenic acid in the roots of Longyou No. 7, a cold-resistant variety, were 157% and 89% higher, respectively. This indicated that cold-resistant varieties exhibit significantly higher levels of linoleic acid, linolenic acid, and IUFA under low-temperature stress. Li et al. [48] further confirmed that the content of linoleic acid and linolenic acid in safflower stems gradually increased after low-temperature treatment. The biosynthesis of jasmonic acid relies on alpha-linolenic acid, which is released from the galactolipids of the chloroplast membrane [49]. Numerous studies have demonstrated that jasmonic acid enhances the ability of plants to withstand low temperatures [50,51,52].

Flavonoids, a major class of secondary metabolites, are synthesized by plants during long-term ecological adaptation to counteract various stresses, including harsh environmental conditions, herbivores, and microbial attacks [53]. Consistent with the findings of Janas et al. [54], the flavonoid content in soybean roots significantly increased after 24 h of low-temperature cultivation. However, when the competitive inhibitor of phenylalanine ammonia-lyase (PAL), aminoindanphosphoric acid (AIP), was introduced, the flavonoid levels decreased. These results suggest that low temperatures can enhance flavonoid metabolism in plants, thereby improving their adaptability to cold stress. Additionally, under high-temperature stress, grapefruits synthesize substantial amounts of salicylic acid (SA), which induces PAL activity and increases the flavonoid content [46]. This indicates that flavonoids also play a critical role in regulating heat stress resistance. Luo et al. [55] transplanted plants from high-latitude to low-latitude regions and cultivated them under artificially simulated ultraviolet (UV) radiation. They observed an exponential increase in flavonoid content in plant leaves, demonstrating the protective role of flavonoids against UV radiation. Leng et al. [56] investigated the relationship between flavonoids in persimmon branches and cold resistance, revealing that low-temperature stress during autumn and winter leads to the accumulation of reactive oxygen species (ROS) in persimmon branches. Flavonoids can scavenge oxygen ions and other free radicals, thereby mitigating the damage caused by low-temperature stress. Studies have also shown that plants deficient in flavonoids are highly susceptible to environmental stress [57]. Zhou Ping [58] reported a significant increase in flavonols and flavonoid-related derivatives in peach leaves under low-temperature stress. Similarly, Xu Yuan et al. [59] observed that as winter temperatures decreased, the cold resistance of *Rosa serrata* branches gradually increased. This was accompanied by enhanced starch and sugar metabolism, continuous upregulation of flavonoid biosynthesis, and significant increases in soluble sugars and flavonoid content. Additionally, proteins related to the antioxidant system, cell wall relaxation and elongation, water transport channels, and cell dehydration tolerance were significantly upregulated.

We also identified that the metabolism of α-linolenic acid, which has been previously confirmed in pumpkin rootstock varieties [60], along with phenylpropanoid biosynthesis, flavonoid biosynthesis, and phenylalanine metabolism, as the most active pathways under chilling stress. This suggests that unsaturated fatty acids, phenylpropanes, and flavonoids (including flavonoid metabolites) play a crucial role in walnut’s response to chilling stress. Such as 109013393 was significantly negatively correlated with methyl dihydrojasmonate and methyl jasmonate, 109018148 was significantly positively correlated with L-phenylalanine and phenethylamine, 109010746 was significantly positively correlated with laricitrin, 108993196 was significantly positively correlated with neohesperidin and ferulaldehyde, 108989769 was positively correlated with neohesperidin, rutin, and hesperetin and significantly negatively correlated with eugenol, 109020389 was significantly positively correlated with L-phenylalanine and ferulaldehyde, 109020701 was highly significantly positively correlated with L-phenylalanine and significantly positively correlated with ferulaldehyde, 109003193 was significantly positively correlated with coniferin, 108999621 and 109004171 were significantly negatively correlated with ferulaldehyde, 109009576 and 108983554 were significantly positively correlated with ferulaldehyde, with the latter also showing a significant positive correlation with L-phenylalanine, 109005199 was significantly positively correlated with coniferin, 108996382 was significantly negatively correlated with L-phenylalanine and ferulaldehyde, and soon. These genes can serve as potential targets for walnut breeding, and these findings provide valuable insights into the regulatory relationships between genes and metabolites, offering a theoretical foundation for further research on gene-mediated metabolic regulation in response to cold stress. This study elucidates the synergistic mechanisms of unsaturated fatty acids, phenylpropanoids, and flavonoids of walnuts’adaptation in chilling stress. Notably, unsaturated fatty acids (e.g., linoleic and α-linolenic acids) constitute up to 91% of walnut fatty acids, with their increased desaturation lowering membrane phase transition temperatures to preserve fluidity under cold conditions—a process potentially regulated by cold-induced *FAD2/FAD3* genes, as observed in Arabidopsis [61]. Environmental factors like altitude and accumulated temperature significantly influence fatty acid composition, with high-altitude conditions promoting polyunsaturated fatty acid accumulation while balancing lipid peroxidation risks [62]. Concurrently, phenylpropanoid metabolism exhibits dual roles: lignin deposition via upregulated *PAL/C4H* enhances cell wall integrity, while coumaric acid derivatives scavenge ROS, mirroring cold adaptation strategies in apples [63]. Flavonoid spatiotemporal specialization is evident, as root-specific phloridzin chelates radicals, whereas leaf flavonol glycosides protect photosynthetic systems via UV absorption, potentially linked to UFGT glycosyltransferase localization [64]. These pathways share phenylalanine precursors, with cold-induced carbon reallocation redirecting metabolic flux toward defense compounds. Crucially, flavonoid intermediates (e.g., naringenin) may interact with jasmonic acid signaling, forming a metabolite-defense network. While identifying molecular markers (e.g., FAD3 SNPs) aids cold-resistant cultivar breeding, excessive desaturation-induced peroxidation necessitates caution. Future studies should integrate lipidomic–transcriptomic analyses to delineate antioxidant-desaturation crosstalk and validate key gene functions (e.g., ANS) using transgenic models while optimizing cultivation strategies based on ecological factors influencing α-linolenic acid synthesis. Based on the KEGG annotation pathway database, this study proposes a concise metabolite network.

Currently, metabolomics alone is insufficient to fully elucidate the comprehensive response mechanisms of a species. In subsequent research, we plan to integrate transcriptomics, proteomics, and other omics approaches to further unravel the molecular mechanisms underlying walnut’s response to chilling stress.

## 5. Conclusions

This study elucidates the dynamic metabolic reprogramming of walnut leaves under chilling stress through comprehensive LC-MS/MS-based metabolomic profiling. By identifying 1504 metabolites and characterizing cultivar-specific differential metabolites in ‘Qingxiang’ and ‘Liaoning No.8’, we revealed critical pathways, including α-linolenic acid metabolism, phenylpropanoid biosynthesis, and flavonoid biosynthesis, as central components of the walnut’s adaptive response to low-temperature stress. The significant correlation between candidate genes and key metabolites underscores the molecular-metabolic interplay underlying cold tolerance. Furthermore, the proposed metabolic network offers valuable insights for molecular breeding strategies aimed at enhancing cold resistance in walnut cultivars, thereby addressing ecological and agricultural challenges posed by chilling environments.

## Figures and Tables

**Table 1:** Differential screening results of metabolites.

Compared Samples	Total Identified	Total Significantly Changed	Significantly Upregulated	Significantly Downregulated
QXT vs. QXCK positive	871	160	33	127
L8T vs. L8CK positive	871	287	233	54
QXT vs. QXCK negative	633	83	37	46
L8T vs. L8CK negative	633	206	114	92

## Data Availability

No new data were created or analyzed in this study.
