# Correction: Liu et al. Metabolite Profile and Metabolic Network Analysis of Walnuts (*Juglans regia* L.) in Response to Chilling Stress. *Metabolites* 2025, *15*, 394

**DOI:** 10.3390/metabo16030186

**Published:** 2026-03-10

**Authors:** Kai Liu, Yang Li, Yaxin Sang, Yaru Zhang, Xiuhong An, Hongxia Wang, Ruifen Zhang

**Affiliations:** 1 Scientific Research Office, BinZhou Polytechnic, Binzhou 256603, China; liukai@bzpt.edu.cn (K.L.); liyang902102@163.com (Y.L.); 15615534384@163.com (Y.Z.); 2 College of Food Science and Technology, Hebei Agricultural University, Baoding 071000, China; sangyaxin@sina.com; 3 College of Horticulture, Hebei Agricultural University, Baoding 071000, China; anxiuhong2007@126.com; 4 Qingdao Academy of Agricultural Sciences, Qingdao 266000, China

The authors would like to make the following correction to their published paper [1]. The change is as follows:

Reference [21] in the original publication is incorrectly cited and needs to be replaced with the following reference.

21. Zhang, Y.; Dan, Q.; Zhou, D.; Yan, Y.; Pan, L.; Tu, K. Low-temperature storage affects the quality and antioxidant capacity of “Xiahui No. 8” peach fruit: Mechanisms involving the regulation of the antioxidant system and pentose phosphate pathway. *Postharvest Biol. Technol.* **2026**, *234*, 114098.

The authors state that the scientific conclusions are unaffected. This correction was approved by the Academic Editor. The original publication has also been updated.
